# Persistence of collective memory of corporate bankruptcy events discussed on X (Twitter) is influenced by pre-bankruptcy public attention

**DOI:** 10.1038/s41598-024-53758-x

**Published:** 2024-03-19

**Authors:** Kathleen M. Jagodnik, Sharon Dekel, Alon Bartal

**Affiliations:** 1 The School of Business Administration, Bar-Ilan University, Ramat Gan, 5290002 Israel; 2 Department of Psychiatry, Harvard Medical School, Boston, MA 02129-4522 USA; 3 Department of Psychiatry, Massachusetts General Hospital, Boston, MA 02129-4522 USA

**Keywords:** Bankruptcy, Collective memory, Dynamic events, Public attention, Social media, Computational biology and bioinformatics, Computational models, Data mining

## Abstract

Collective attention and memory involving significant events can be quantitatively studied via social media data. Previous studies analyzed user attention to discrete events that do not change post-event, and assume universal public attention patterns. However, dynamic events with ongoing updates are common, yielding varied individual attention patterns. We explore memory of U.S. companies filing Chapter 11 bankruptcy and being mentioned on X (formerly Twitter). Unlike discrete events, Chapter 11 entails ongoing financial changes as the company typically remains operational, influencing post-event attention dynamics. We collected 248,936 X mentions for 74 companies before and after each bankruptcy. Attention surged after bankruptcy, with distinct Low and High persistence levels compared with pre-bankruptcy attention. The two tweeting patterns were modeled using biexponential models, successfully predicting (F1-score: 0.81) post-bankruptcy attention persistence. Studying bankruptcy events on social media reveals diverse attention patterns, demonstrates how pre-bankruptcy attention affects post-bankruptcy recollection, and provides insights into memory of dynamic events.

## Introduction

Collective memory encompasses significant events shared by social groups and manifests in both communicative (individual-level communications) and cultural forms (societal activities such as rites and monuments)^1–3^. Psychologists explore memory formation and retention through top–down approaches (focusing on familiarity, narrative templates, and cultural attractors) and bottom-up approaches (concentrating on retrieval-induced forgetting and social affinities)^4–8^.

Computational social scientists quantify collective memory through indicators including number of discussions online, Wikipedia page views, and citations of academic papers, which reflect public attention to cultural products^3,9–12^. However, this approach, while insightful, does not directly capture the essence of collective memory but rather tracks the spillovers of public attention that influence memory formation^3^.

Cultural products initially receive high attention close to their release (communicative memory), which diminishes over time to cultural memory^3,10^. When a cultural product becomes popular, people discuss it widely (high attention). This heightened interest makes individuals more likely to remember a product. When a cultural product moves away from communicative memory, it loses this intense attention. Hence, attention has a vital role in the formation and dissemination of collective memory^3^. The computational approach aligns closely with Jan Assmann’s^13^ definition of collective memory, which focuses on the cultural products remembered by communities or groups of people. In this work, we follow the computational social science approach to study collective memories of significant events by analyzing cultural consumption of information expressed by attention. Similar to previous studies^3,9–12^, this study operates under the assumption that the frequency of event mentions on social media is proportional to the level of public attention or collective memory.

Attention plays a critical role in the persistence of collective memory. Significant events, especially those linked with corporate irresponsibility like bankruptcy, exhibit varying levels of persistence in collective memory, influenced by factors such as media representation and company strategies^14–16^. Companies often employ short-term and long-term strategies to shape or erase the memory of negative events, ranging from influencing media narratives to silencing detractors^17–22^.

Recently, empirical social media data have been employed to measure the persistence of collective memory, using data sources including Wikipedia page views and edit histories of pages related to significant events, e.g., disasters, accidents, and attacks^23–25^. Researchers have modeled the persistence of collective memory using various decay functions. An exponential model was fit to describe the daily decay of Wikipedia page views on aviation accidents^26^. The stretched exponential model was used to describe the daily page views of online academic articles, showing an initial rapid decay followed by a slower decay rate^27^. Candia et al.^3^ employed a biexponential function (*C*_1_*e*^−*αt*^ + *C*_2_*e*^−*βt*^) to describe both communicative and cultural memory aspects in the citations of publications and patents, and in online attention to songs, movies, and athletes’ biographies on Wikipedia. Furthermore, the shifted power-law function (*C*_1_*t*^−*α*^ + *C*_2_) has been utilized to model the persistence of memory of deceased celebrities via news page views and X (formerly Twitter) mentions^10^.

Strategies to influence collective memory can revise persistence levels of the event’s memory^14^, leading to positive or negative organizational outcomes. Successful corporate strategies can reduce negative communication of a bankruptcy event^28^. Furthermore, slow information spread of news about a company’s financial difficulties has been found to enhance the effectiveness of momentum-based strategies in facilitating the company’s recovery^29,30^. These findings highlight the importance of analyzing temporal communication in managing the recovery, and the persistence level, of distressed companies in collective memory.

Despite recent progress, the theory of collective memory and persistence of public attention lacks quantitative models that analyze empirical data. Earlier studies on collective memory mainly focused on events such as publishing a new song, movie, or research article; as well as events documented in Wikipedia, such as natural and human-generated disasters, accidents, terrorism attacks, anniversaries, or commemorative events^3,9–12,23^. These events are *discrete* in the sense that they are unalterable after taking place. We extend previous work by analyzing *dynamic* events of bankrupt companies that remain operational and continue to evolve financially following bankruptcy. Previous studies have also largely assumed that the persistence level of attention to an event is similar across individuals^3,10,31^. However, memories of a past event often differ among individuals^14^, implying different attention levels. This conflict in the literature raises the need for studying collective memory of dynamic events with diverse persistence attention levels, addressed by this study.

In this study, we extend the literature by studying how attention to corporate bankruptcy events evolves over time using hundreds of thousands of posts (tweets) from the social media platform X that mention U.S. companies that filed for Chapter 11 bankruptcy from 2012 through 2022. Under Chapter 11, companies can sustain their financial operations. We constructed the time series for each company by tracking its daily mention frequency on X for a 30-day window before and after its bankruptcy declaration. This 30-day period was chosen to adequately capture the immediate surge in public attention surrounding the bankruptcy event (communicative memory), as well as the subsequent transition to a more stable, long-term pattern of mentions (cultural memory), thus providing a broad view of the evolving collective memory related to these bankruptcy events. Whereas most previous studies about bankruptcy explored stock performance, either in the short term following the announcement (e.g., during the 10 days post-bankruptcy^32^), or in the medium-term (e.g., analyzing the monthly Chapter 11 stock performance of 56 firms^33^), we innovate by analyzing post-bankruptcy memory for 1 month following bankruptcy declaration via daily time series analysis of mention frequency before and after bankruptcy. More specifically, we employ sophisticated time series analytical techniques, including the fitting of five distinct collective memory models – biexponential, exponential, hyperbolic, logarithmic, and power models – to the observed mention frequencies, allowing us to accurately characterize and quantify the dynamics of collective memory in both the short-term and long-term phases following a company’s bankruptcy announcement.

For each bankruptcy event, we estimated model parameters separately for Low- and High-persistence level of collective memory. This involved determining the initial communicative memory strength and the rates of decay for both the immediate and prolonged phases of attention. Using the Akaike Information Criterion (AIC) and R-squared values, we assessed the fit of each model to the empirical data. The decay patterns observed through these models provide insights into how public attention to bankruptcy events dissipates over time. By fitting these models and analyzing their parameters, we infer not just the intensity of public attention following bankruptcy announcements but also understand the longevity and depth of this attention in collective memory.

This approach allows us to explore the dynamics of public interest and sentiment towards corporate bankruptcy in a more structured and quantifiable manner, leading to the formulation of three hypotheses:

### H1

Low versus High levels of persistence in collective memory have different temporal decaying patterns of attention following bankruptcy announcement.

To address *H1*, we quantify the increase and decay of attention following bankruptcy announcement, and observe decaying patterns associated with Low or High persistence level (defined in the Methods section) of collective memory. We fit five decay models for each of the two observed patterns of persistence attention (Low and High) that are best captured by two biexponential functions.

### H2

Low versus High levels of persistence in collective memory present different user attention patterns, starting at an early stage before bankruptcy announcement.

To address *H*2, we analyze the variations in societal memory persistence level of bankrupt companies.

### H3

The persistence level of a bankruptcy-related memory event can be predicted at bankruptcy announcement day.

To address *H3*, we identify differences in attention before bankruptcy and predict, on bankruptcy day, the post-bankruptcy persistence level of collective memory.

## Results

### Memory of companies following bankruptcy

**Figure 1:**
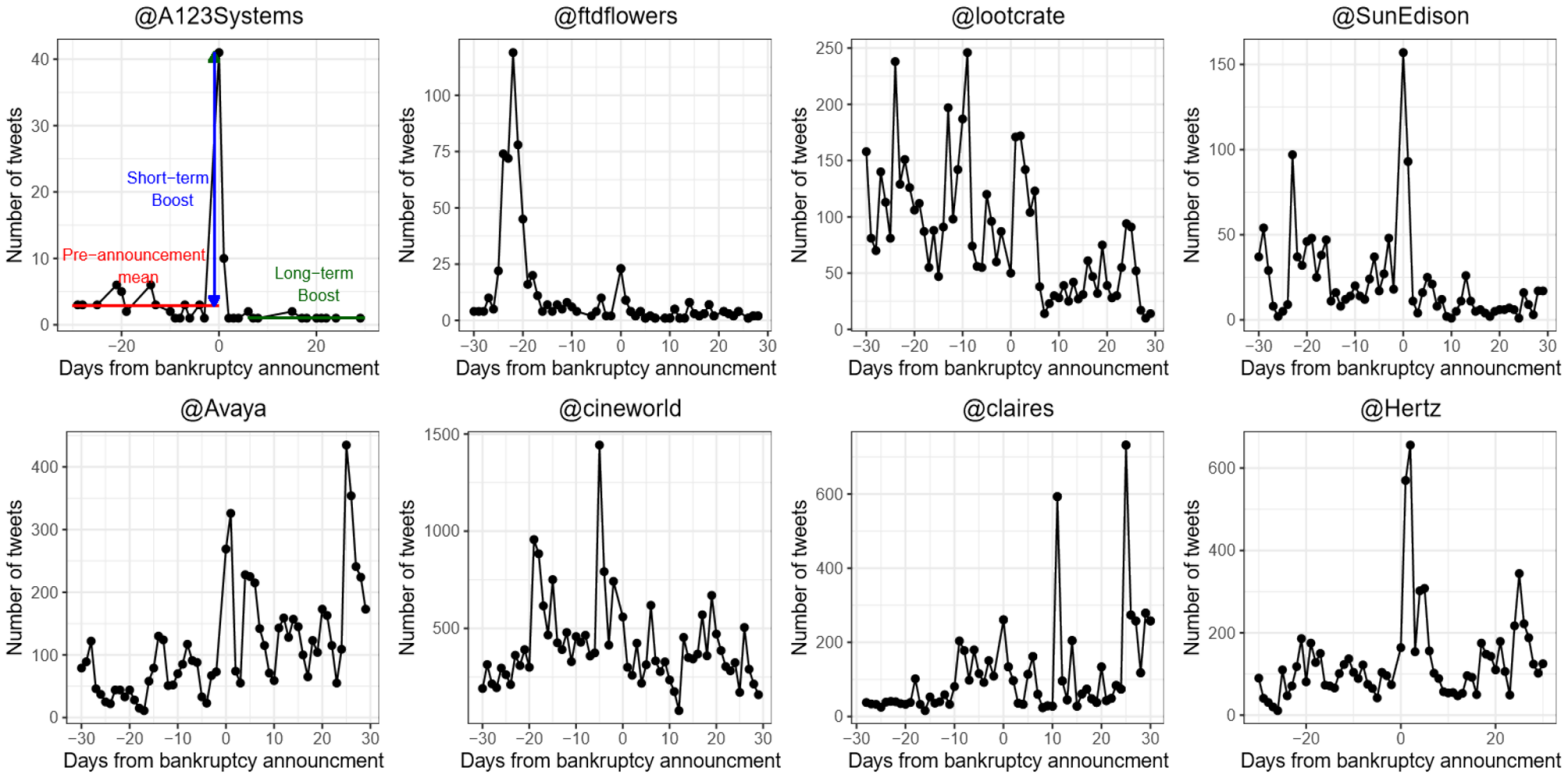
Examples of mention time series for 8 companies that declared Chapter 11 bankruptcy, as recorded from X posts. Companies’ X handles begin with ‘@’ above each figure. Companies in the top row experienced a rapid increase followed by a quick decay in attention after bankruptcy announcement at *t*_0_. Companies in the bottom row also experienced a rapid increase in attention following bankruptcy, but attention was more persistent (decreased more slowly) compared with companies in the top row in terms of days until returning to the mean daily mentions before bankruptcy announcement (Pre-Announcement Mean). From each mention time series, we extract three characteristic values (illustrated in the top left panel): Pre-Announcement Mean (PAM), Short-Term Boost, and Long-Term Boost, as defined in Table 1.

Using the collected X posts (tweets), we characterize the patterns by which public attention evolved before (during the month prior to) and after (during the month following) bankruptcy announcements of 74 U.S. companies that declared Chapter 11 bankruptcy. Our analysis focuses on time series of company mention frequency. The raw mention time series of company *i* specifies the number of tweets mentioning company *i* on each day *t* relative to *i*’s bankruptcy announcement day (*t*_0_). Pre- and post-bankruptcy time series were computed for each company (Fig. 1).

**Figure 2:**
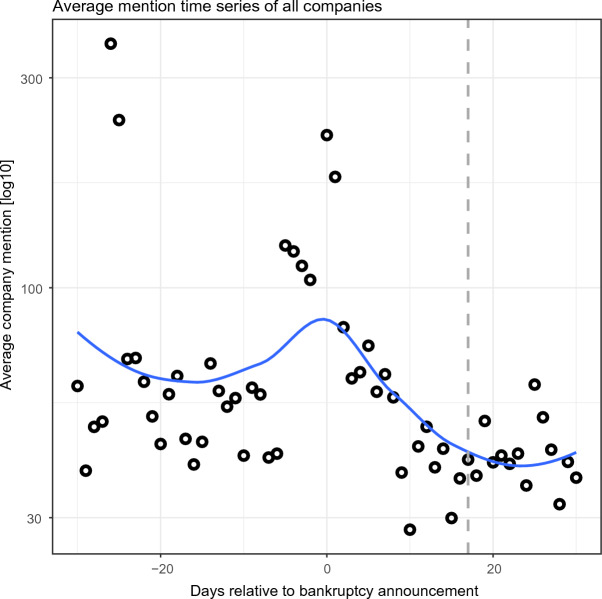
Average company mentions time series of the 74 companies that declared Chapter 11 bankruptcy that were included in the study (see Fig. 1 for examples of individual mention time series). The average was obtained via the arithmetic mean of the individual raw mention time series of companies that declared bankruptcy. The average mention frequency value spikes at the day of bankruptcy announcement (*t*_0_) and fades quickly after *t*_*I*_ = 17 days (vertical dashed line). Inflection Point *t*_*I*_ denotes the maximum curvature cutoff point for *t* > 0. Circles correspond to raw average mention time series, and the blue curve, to the mean smoothed version.

The averaged raw mention time series of companies that declared bankruptcy, reflecting the tweets of 132,748 X users, are presented in Fig. 2. We observe (Fig. 2) a pronounced spike in public attention to companies on the day of bankruptcy announcement (*t*_0_). This spike is followed by a precipitous drop until day 17 after the announcement (vertical dashed line, Inflection Point *t*_*I*_ = 17 in Fig. 2), when the curve shifts into an extended, flatter phase. The Inflection Point (*t*_*I*_) was found using the KneeArrower R library by locating the point of maximum curvature. This point is a mathematical measure to identify a ‘knee’ in a curve^34^. The value of *t*_*I*_ indicates the time when communicative memory substantially levels off.

Additionally, we observe (Fig. 2) a spike in average public attention at *t* = − 26*,* − 25. These spikes result from high attention to the Cumulus Media company, with 13,656 mentions in these 2 days, increasing the average attention to 371.6 and 257.76 for *t*_−26_ and *t*_−25_, respectively. The timestamps of *t*_−26_ and *t*_−25_ correspond to November 4th and 5th, 2017, before Cumulus Media announced bankruptcy on November 29, 2017. These spikes in attention during the 30 days before bankruptcy announcement are observed for some of the other companies, as well (Fig. 1). These shifts in attention serve as the motivation for *H*_1_ and *H*_2_, the testing of which will be described in subsequent sections.

Observing the period before bankruptcy, a company’s baseline cultural memory level can be represented by the Pre-Announcement Mean (PAM) value (Table 1). PAM is a constant value measuring mention frequency on social media, observed at *t* < *t*_0_. A bankruptcy event causes a burst of quickly fading short-term communicative memory (Short-Term Boost) at *t* ∈ [*t*_0_*,t*_*I*_ = 17], layered upon this baseline level.

**Table 1:** Definitions of variables in this study.

Parameter	Definition	Mathematical Definition
Bankruptcy event start	Day of bankruptcy announcement	*t*_0_
Studied attention period	Time period (∆t) in days, before and after bankruptcy announcement	*t* ∈ [*t*_0_ − ∆*t,t*_0_ + ∆*t*]
Pre-Announcement Mean (PAM)	Mean daily mentions before bankruptcy announcement	*Mean*({*mentions*_*t*_|*t* < *t*_0_})
Inflection point (*t*_*I*_)	Point in time when communicative memory shifts into an extended, flatter phase	{*t*_*I*_|mentions_*tI*_ ≤ *PAM,t*_*I*_ > *t*_0_}
Short-Term	Time period (days) between bankruptcy announcement and *t*_*I*_	*t* ∈ [*t*_0_*,t*_*I*_]
Long-Term	Time period (days) after *t*_*I*_	*t* > *t*_*I*_
Short-Term Boost	Maximum daily mentions in the Short-Term period minus PAM	*Max*({*mentions*_*t*_|*t* ∈ [*t*_0_*,t*_*I*_]}) − *PAM*
Long-Term Boost	Mean daily mentions after the Inflection Point minus PAM	*Mean*({*mentions*_*t*_|*t* > *t*_*I*_}) − *PAM*
Persistence level of memory	Maximum daily mentions in the Long-Term period compared with PAM	*High* : *if Max*{*mentions*_*t*_ | *t* > *t*_*I*_} > PAM *Low* : otherwise

However, we observe (Fig. 2) that while collective memory quickly returns to approximately the PAM volume when averaging over all companies, this is not consistently true for individual companies (Fig. 1). Therefore, we divide the bankruptcy post-announcement period into Short-Term (*t* ∈ [*t*_0_*,t*_*I*_ = 17]) and Long-Term (*t* ∈ (18*,*30]) phases (Table 1). Using these two phases, we next analyze the shape of X mentions time series via three characteristic values (Table 1): Short-Term Boost, Long-Term Boost, and PAM.

#### Magnitude of Short- and Long-Term Boosts in attention

For each company mention series during *t* < *t*_0_, we removed outliers using the R boxplot function, as they can impact PAM, which approximates a company’s cultural memory. SI Appendix A1 (Methods) elaborates how we identified and removed outliers. The mean PAM was 31.83 (95% CI [11.81, 51.85]). The mean Short-Term Boost was 210.08 (95% CI [94.16, 325.99]). On average, a bankruptcy event spikes attention by ∼560% ([210.08-31.83]/31.83). The mean Long-Term Boost was 58.73 (95% CI [28.31, 89.16]). Users’ interest on X typically rapidly faded after announcement. Some companies have larger Long-Term Boosts than others, implying that some bankruptcy events persist longer in collective memory. This can be observed in Fig. A1 that presents the distribution of Long-Term Boost.

Accordingly, we define Low and High levels of persistent memory (Table 1). Then, we identify the persistent memory level of each bankruptcy event (noted by company name) as Low or High. We found 26 and 48 companies with Low and High levels of bankruptcy memory persistence, respectively, as observed in Fig. A2 that presents histograms of companies with: (a) Low, or (b) High persistence level of memory. These two persistence levels imply different temporal decaying patterns of attention following bankruptcy (*H*_1_).

### Memory model fitting

**Figure 3:**
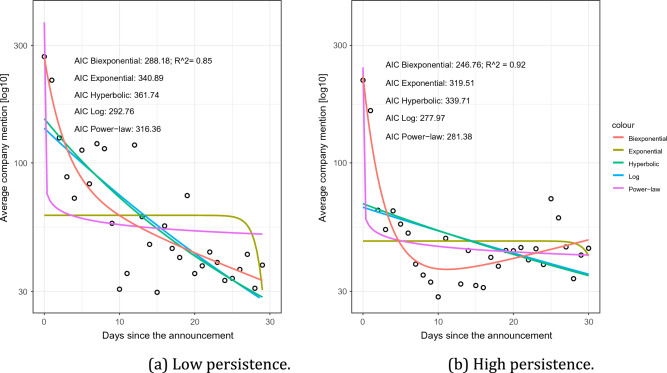
Model fitting for corporate bankruptcy events of Low and High memory persistence. Higher *R*^2^ and lower AIC values indicate a better fit.

To test *H*_1_, we fit five mathematical models to the post-bankruptcy empirical average mention time series. More specifically, we performed model fitting for the two time-series datasets *S*(*t*) of (i) Low, and (ii) High persistence of memory. In model fitting, we added a constant value *ε* to each time series, where *ε* was the minimum nonzero value across all individual time series. Then, we took base-10 logarithms of the empirical data and conducted parameter fitting of each model formula to the data in a log–log space using a nonlinear least-squares method^10^. We compared the performances of the models using *R*^2^ and AIC of each model for each Low (Fig. 3a) and High (Fig. 3b) level of persistent memory. The Biexponential model best fits the empirical average mention time series for both the initial exponential decay, and the longer, slower decay for the Low (*R*^2^ = 0.85;* AIC* = 288.18) and High (*R*^2^ = 0.92;* AIC* = 246.76) levels of persistent memory (Fig. 3).

Fig. 3 presents the different shapes of the fitted Biexponential curves for the Low and High levels of persistent memory. The Biexponential model *S*(*t*) is composed of (i) communicative memory *u*(*t*) = *Ne*^−(*p*+*r*)*t*^, and (ii) cultural memory *v*(*t*) = *Nr*[(*e*^−*qt*^ − *e*^−(*p*+*r*)*t*^]. Its fitted parameters *S*(*t*) = *a*_1_*e*^−*b*1∗*x*^ + *a*_2_*e*^−*b*2∗*x*^ for companies with Low (*a*_1_ = 158.98, *b*_1_ = 0.76, *a*_2_ = 109.12,* b*_2_ = 0.04) or High (*a*_1_ = 183.47, *b*_1_ = 0.54, *a*_2_ = 28.61, *b*_2_ =  − 0.01) level of persistent memory significantly differ. SI Appendix A4 (Memory Model Fitting) provides the computational code for model fitting for companies in each memory persistence level (Low and High). In terms of the Biexponential model, *u*(*t*_0_)=*N,* the parameters *a*_1_ and *b*_1_ are related to the decay rate of communicative memory (*r*) and the time (*t*), while *a*_2_ and *b*_2_ are related to the decay rate of cultural memory (*q*) and the time (*t*). Specifically, *a*_1_ = *p/*(*p* + *r*) and *b*_1_ = *ln*(2)*/*(*t* ∗ *ln*(*p* + *r*)*/p*), while *a*_2_ = *q/*(*q* + *r*) and *b*_2_ = *ln*(2)*/*(*t* ∗ *ln*(*q* + *r*)*/q*).

The various significant parameter values found for Low versus High persistent memory show distinct patterns of user attention following bankruptcy announcement, supporting *H*_1_.

### Detecting memory persistence level in early stages (pre-bankruptcy)

To test *H*_2_, we examine whether Low and High levels of persistent memory are characterized by different attention patterns (mentions) to companies *before* and up to bankruptcy day (*t* ≤ *t*_0_).

**Figure 4:**
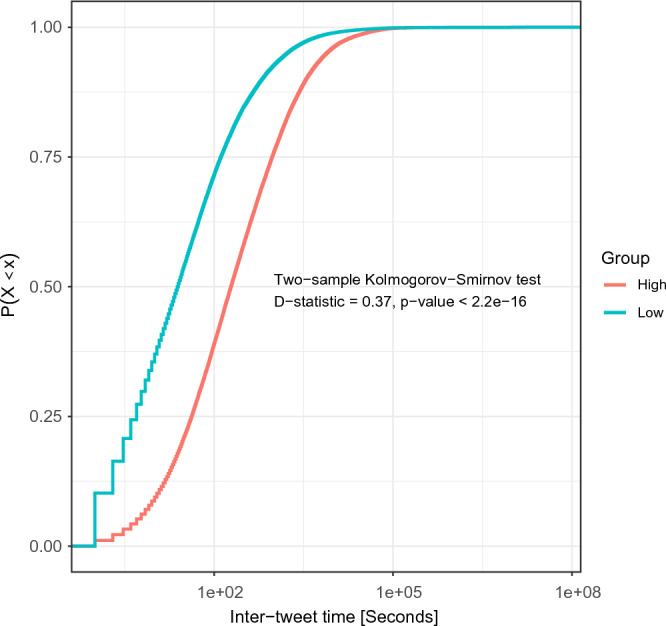
Empirical Cumulative Distribution Function (ECDF) of inter-tweet time before bankruptcy announcement for Low and High persistent memory of corporate bankruptcy events. Low and High persistent memory have different temporal tweeting patterns, identified via comparison of the two ECDFs by using the Kolmogorov–Smirnov test. *P*-value < 0.05 indicates that the samples are not drawn from the same distribution.

To analyze attention patterns, for each company we calculate the time difference between every two consecutive tweets (i.e., inter-tweet times) that mention a company with either Low or High level of persistent memory. Figure 4 presents two Empirical Cumulative Distribution Functions (ECDFs) of inter-tweet times that correspond to each memory persistence level.

We compare the distributions of the ECDFs in Fig. 4 by using the Kolmogorov–Smirnov (KS) D-statistic test^35^. The D-statistic is defined as the maximum distance: *D* = *max*(|*F*1(*x*) − *F*2(*x*)|), where *x* represents the range of the random variable, and F1 and F2 represent the ECDFs. The smaller the distance, the more similar the distribution curves and, hence, the more likely are the two samples to have come from the same distribution. The KS test shows significant differences (*D* = 0.37; *P*-value < 2.2 × 10^−16^) between the ECDFs. Thus, temporal user attention patterns to events with Low versus High levels of persistent memory significantly differ at early stages *before* bankruptcy announcement, supporting *H*_2_.

The ECDF curve (Fig. 4) for Low level of persistent memory is located above the ECDF of High level of persistent memory. This indicates that users are more likely to mention Low-persistence memory events. Additionally, the time needed to reach 75% of inter-tweet times is longer for High versus Low level of persistent memory. In other words, information on bankruptcy events of High memory persistence spreads more slowly but endures longer than information on bankruptcy events of Low memory persistence.

Successful corporate strategies reduce negative communication of a bankruptcy event^28^. Momentum-based strategies are more effective for a distressed company when its financial information spreads more slowly^29,30^. If a successful strategy were implemented in a firm, it would be expected that the memory of events with High persistence (slower information spread) would exhibit less negative sentiment compared with the memory of events with Low persistence.

#### Sentiment differences in low versus high collective memory

Using the VADER Python library^36^, we analyze tweet sentiment for events with Low or High persistence before and after corporate bankruptcy announcement.

We computed the overall compound score (All) for each tweet, which represents a normalized combination of positive, negative, and neutral lexicon ratings ranging from − 1 (extremely negative) to + 1 (extremely positive). However, this approach has a drawback: tweets with a mix of strongly positive and strongly negative sentiments can receive similar scores to tweets containing neutral terms. To overcome this drawback, we calculated the positive (Pos), negative (Neg), and neutral (Neu) scores for each tweet. These three scores are ratios for proportions of text that is grouped in each category.

Using VADER, the Neg score represents the negative sentiment of a text, ranging from 0 (no negativity) to 1 (highest negativity). Pos and Neu scores follow similar logic. Boxplots of sentiment values before and after bankruptcy are presented for Low (Fig. A3a) and High (Fig. A3b) persistence levels.

We performed pairwise Wilcoxon tests to identify significant differences between the sentiments of events with Low or High persistence. Events with Low persistence (Fig. A3a) show significant differences (*P*-values = 5.921*e*^−4^, 0.018, and 1.198*e*^−3^) between the median sentiment before (0.109, 0.039, and 0.128) versus after (0.089, 0.051, and 0.081) bankruptcy announcement of Pos, Neg, and All, respectively. Events with High persistence (Fig. A3b) show significant differences (*P*-values of 3039*e*^−6^, 3.297*e*^−5^, 0.012, and 4.832*e*^−8^) between the median sentiment before (0.125, 0.032, 0.824, and 0.225) versus after (0.113, 0.039, 0.830, and 0.171) bankruptcy announcement of Pos, Neg, Neu, and All, respectively.

Next, we tested whether the increase of Neg sentiment within Low-persistence events is significantly different than that of High-persistence events. We computed the percentile decrease (*δ*) of Neg sentiment before versus after bankruptcy for both Low (*δ* = 119%) and High (*δ* = 117.17%) persistence levels. We found significant differences (*P*-value = 8.038*e*^−9^) in *δ* of Neg sentiment for Low versus High persistence. Similarly, we found significant differences in *δ* of Neu sentiment (*P*-value = 1.768*e*^−6^) of Low (*δ* = 100.69%) versus High (*δ* = 101.02%) persistence. These findings show that (i) the percentile increase in Neg sentiment before versus after bankruptcy is significantly smaller for High compared with Low memory persistence; and (ii) the increase in Neu sentiment before versus after bankruptcy is significantly higher for High- compared with Low-persistence memory. Figure A3 presents boxplots of sentiment before and after bankruptcy announcement in the Low and High memory persistence levels. This finding implies that companies with High-persistence memory, having slower information spread (Fig. 4), may have employed a successful bankruptcy strategy, as they exhibit significantly lower percentile increase of negative (Neg) sentiments.

In the next section, we explain the persistence level of the memory about a corporate bankruptcy event, whether Low or High.

### Explaining memory persistence

To test *H*_3_, given a bankruptcy event, we aim to predict, using a logistic regression model, whether its memory will gain low or high user attention, i.e., will have Low or High persistence. We measured the following explanatory terms for each bankruptcy event.

1. Avg time from first—Average time difference between pre-event tweets and the first tweet mentioning the bankruptcy event for *t* ≤ *t*_0_. Captures bursty user interactions that can explain tweeting behavior^37^.
2. Pre announcement mean (PAM)—Cultural memory (Table 1).
3. Followers—Number of users who subscribed to receive updates from a company’s X account.
4. Following—Number of X accounts subscribed to by a company.
5. Short-term boost (Table 1)—Attention boost during days *t*_0_ to *t* = 17.
6. Number of tweets before—Number of tweets before bankruptcy at *t* < *t*_0_.
7. Avg txt len—Average tweet length (posted at *t* < *t*_0_).
8. Avg sentiment—Average tweet sentiment (ranges between − 1 and 1).
9. Private public—Whether a company was publicly traded or privately held on day of bankruptcy announcement.

We define a binary outcome variable to be Low (*y* = 1) or High (*y* = 0) persistence level. A backward stepwise logistic regression found Terms #1, #2, and #6 significant. SI Appendix A6 (Persistence Prediction) provides the results of the developed logistic regression for binary classification, including evaluation performances. Term #1: A negative coefficient of − 0.23 indicates that, on average, the greater the time difference between the posting of a given tweet and the first tweet mentioning the bankruptcy event, the more likely the memory of a bankruptcy event will have High persistence. Term #2: A negative coefficient of − 0.13 indicates that the larger the PAM, the more likely an event will gain High persistence. Term #6: A positive coefficient of 0.3*e*^−2^ indicates that the more a company is mentioned before bankruptcy, the more likely its bankruptcy event will gain Low-persistence memory.

#### Estimating model performance

We estimated model performance in predicting whether a bankruptcy event will experience Low or High persistence, using the F1-score (values closer to 1.0 indicate better performance). We used 70% of the data for training, and 30% for testing our model. A ten-fold cross-validation using the Train set resulted in an average F1-score of 0.87. When we used the Test set, our model yielded F1-score of 0.81 for identifying events with High-persistence memory, providing support for *H*_3_ (SI Appendix A6, Persistence Prediction).

## Materials and methods

### Dataset of bankruptcy events

We opted for the collection and analysis of X data due to several reasons: (i) The platform X draws a vast number of users due to its support of real-time discussions about current events. This attribute of X is particularly valuable for our study as it offers a dynamic perspective on how collective memory evolves; (ii) The extensive and varied user community on X provides a wide range of public opinions and conversations, enriching our dataset; and (iii) The accessibility of X’s API allows for the efficient gathering of substantial data, which is crucial for in-depth analysis. The archival feature of X enables us to conduct a retrospective examination of public sentiments and responses over time, which is essential to comprehend the development of collective memory. Nonetheless, working with X data presents certain challenges and limitations: (i) The demographic characteristics of X users might not be fully representative of the overall population, which could lead to skewed results; (ii) Issues like the presence of automated bots, spam, and the phenomenon of ideological ‘echo chambers’ on X can distort the data; and (iii) The transient nature of tweets and the platform’s data retention policies can restrict longitudinal analyses.

In the context of the current study, X data are advantageous for studying bankruptcy events because they capture immediate, diverse public reactions and sentiments in real time. This contrasts with citation or Wikipedia data, which are more static and less reflective of contemporary public discourse.

We collected a list of 708 U.S.-based companies that filed for Chapter 11 bankruptcy from 2012 through 2022 under the American Bankruptcy Act. Company names and bankruptcy dates were obtained via the S&P Global Market Intelligence (https://www.spglobal.com/marketintelligence/en/) online repository as well as the Wikipedia category pages of companies that filed for Chapter 11 bankruptcy in each year. Manual investigation retrieved 137 companies with X handles that continue to operate.

Using the X API, we collected X posts (tweets) in the English language that mentioned a given company’s X handle for a period of 1 month (∆_*t*_ = 30 days) *before* and 1 month *after* the day of bankruptcy announcement (*t*_0_). To allow meaningful learning of user attention, we discarded companies with the following characteristics: (i) companies that had Followers < 10 (25 companies), and (ii) companies that tweeted < 10 tweets 30 days before or after the announcement (38 companies). This resulted in a set of 74 companies, 37 publicly traded and 37 privately held (Table B1).

We collected thousands of tweets mentioning each company’s X handle. Discarding tweets originated by the 74 companies resulted in the analysis of 248,936 tweets published by 132,748 users between December 19, 2011 and November 10, 2022.

### Methods

In this section, we first define three characteristic values^10^ to describe communicative and cultural memory before and after bankruptcy announcement. Next, we fit five models to the observed attention patterns to characterize collective memory. Finally, we outline our developed methodology for identifying Low and High levels of persistence memory of bankruptcy events. Based on our analysis, we present an innovative approach for predicting the post-bankruptcy persistence level of attention to a company via the X social media platform's attention patterns to the event early, before bankruptcy announcement.

#### Memory of companies following bankruptcy

Collective memory is expected to sharply increase at event start (*t*_0_) corresponding to bankruptcy announcement, reflecting communicative memory^10^. Then, it is expected to rapidly decay to the pre-event (*t* < *t*_0_) level, reflecting cultural memory^10^. For each company, cultural memory was inferred using the averaged pre-event fraction of event tweet mentions^10^.

To characterize communicative and cultural memory, we identify the *Inflection Point* at Time *t*_*I*_ > *t*_0_, when communicative memory levels off to approximately the pre-event (i.e., pre-bankruptcy announcement) average of daily mentioning. More specifically, we divide the bankruptcy post-announcement period into two phases of *Short-Term* and *Long-Term*. Using these two phases, we analyze the shape of X mentions time series via three characteristic values: *Pre-Announcement Mean*, *Short-Term Boost*, and *Long-Term Boost*. We also define two levels of post-bankruptcy memory: *Low persistence* and *High persistence*. Table 1 summarizes term definitions of this study.

#### Memory model fitting

We compare the fit of five collective memory models *S*(*t*) of previous studies^3,12,26,38,39^ for fitting empirical mention frequencies of post-bankruptcy announcement. We quantify goodness of fit for each model using: (i) *R*^2^, computed as the squared correlation between observed and predicted values on the log scale (larger is better); and (ii) the Akaike Information Criterion (AIC; smaller is better).

1. Biexponential: Collective memory is parameterized by initial communicative memory *N*; decay rates *p,q*, and *r* denote information flow rate from communicative to cultural memory, as follows: *S*(*t*) = *N*[(*p* − *q*)*e*^−(*p*+*r*)*t*^ + *re*^−*qt*^]*/*(*p* + *r* − *q*)
2. Exponential: *S*(*t*) = *ae*^−*bt*^
3. Hyperbolic: *S*(*t*) = (*a* + *bt*)^−1^
4. Logarithmic: *S*(*t*) = (*a* − *blog*(*t*))
5. Power: *S*(*t*) = *at*^−*b*^

The motivation for model selection is detailed next. Exponential and logarithmic models^12,38^ assume that attention should be modeled as a combination of preferential attachment (power-law) and time decay. However, there is a lack of consensus regarding the shape of the decay function and its universality across diverse cultural domains. The biexponential model was found to better fit the memory decay of discrete events, compared with exponential and logarithmic models^3^. Further theoretical motivations for using the four functions other than biexponential are given by Rubin and Wenzel^39^. These exponential, hyperbolic, logarithmic, or linear (power) functions share the property of being concave when plotted on log–log axes (i.e., *log*(*S*(*t*)) is a concave function of *log*(*t*)), whereas the empirical curves are convex on log–log axes.

#### Detecting memory persistence level in early stages

Given that memories of a past event vary among individuals^14^, we hypothesize that detecting the characteristics of social media communications early in the bankruptcy timeline can predict the persistence level (Low or High) of a bankruptcy event in the long term.

To identify differences in user attention, we analyze the tweeting patterns of users mentioning bankrupt companies up to the date of the bankruptcy announcement (*t* ≤ *t*_0_). These bankruptcy events generate either Low or High persistence of memory at *t* > *t*_0_. More specifically, for each persistence level, we first calculate the time difference between every two consecutive tweets (inter-tweet times) that mentioned companies with Low or High persistence level. Second, we generate two Empirical Cumulative Distribution Functions (ECDFs) of inter-tweet times, one for each persistence level. Finally, we test for significant differences in tweeting patterns between the ECDFs using the Kolmogorov–Smirnov (KS) D-statistic test^40^.

Using the developed characteristic values (Table 1), we identify, via classification, on the date of bankruptcy announcement (*t*_0_) whether a bankruptcy event will exhibit Low or High persistence level.

## Discussion

Many studies of collective memory are limited by (i) modeling attention to discrete events that are unalterable after they occur; and (ii) assuming that similar events follow a universal decaying function of communicative memory, measured via the assumption of universal attention patterns to an event, which is not true. To overcome these gaps, we analyzed user attention on the social media platform X to dynamic bankruptcy events, accounting for differences in attention patterns. A company’s financial status under Chapter 11 bankruptcy is dynamic, as the company often continues to operate. Contrary to the common assumption that significant events obey a single decaying function of memory, we found two types of decaying functions corresponding to Low and High persistent memory.

The mention frequency for most bankrupt companies spiked by 560%, on average, at announcement day (*t*_0_), compared with the average Pre-Announcement Mean (Table 1). Subsequently (*t* > *t*_0_), the average mention frequency of the companies dropped sharply, with an Inflection Point of 17 days after the announcement. From this point onward, user attention waned and converged towards the Pre-Announcement Mean. This observation is true when averaging over all companies, but it is not valid for most individual companies (Fig. 1).

Therefore, we divided the bankruptcy post-announcement period according to the Inflection Point into (i) Short-Term (*t* ∈ [0*,*17]) and (ii) Long-Term (*t* > 17) periods (Table 1). We identified Low and High persistence levels of user attention to companies, following bankruptcy announcement. To test *H*_1_, that Low versus High persistent memory have different temporal decaying patterns of attention following bankruptcy announcement, we fit the post-bankruptcy announcement decaying patterns using 10 models (5 for each level) as Low or High. The sharp increase followed by a decrease in users’ attention are best represented by the Biexponential model^3^, which includes two components: (i) a function of long-term cultural memory that accumulates as a result of the company’s operations over time, and (ii) a short-term communicative memory that is added on top of the cultural memory and is invoked by the company’s bankruptcy announcement. The Biexponential model best fits both levels of persistent memory, showing significantly different coefficients for Low and High persistence memory, supporting *H*_1_.

Major financial events are typically covered by mainstream media. The market may anticipate the Chapter 11 announcement when it receives signals related to bankruptcy, before the official announcement is made^41^. Such signals can include reporting losses or declining revenues; replacement of key executives (e.g., CEO); rumors or speculations about the company’s financial health, often found on social media^42,43^; or negative news coverage about the company (e.g., lawsuits or product recalls). These early ‘signals’ are reflected in public attention before bankruptcy announcement. Therefore, public attention to some events might start before the announcement. This led us to hypothesize (*H*_2_) that differences in public attention can be identified before bankruptcy announcement. Indeed, a KS-test revealed that Low and High persistent memory have significantly different temporal attention patterns at early stages before bankruptcy announcement, supporting *H*_2_.

Slow information spread about negative corporate events is essential for successful momentum-based firm strategies^29,30^. The slower information spread and smaller increase in the percentile of negative sentiment observed in High-persistence memory events, compared with Low-persistence events, could suggest the effectiveness of a successful strategy employed by firms in the High-persistence group to handle bankruptcy^28^. Unsurprisingly, following a bankruptcy announcement, we observed a significant decrease in positive sentiment and a significant increase in negative sentiment compared with the pre-announcement values, regardless of the memory persistence levels (Low and High). This can be attributed to financial challenges and potential failure to meet obligations, eroding positive sentiment; and stock market and financial markets reacting to bankruptcy announcements.

Lastly, we successfully (F1-score = 0.81) predicted event persistence level as Low or High in collective memory, supporting *H*_3_. Logistic regression results show that: First, the larger the time gap between the posting of a new tweet and the first tweet mentioning the bankruptcy event (Term #1), the more likely the event will persist longer. This finding is consistent with our results of the ECDF analysis that information on High-persistence bankruptcy events spreads more slowly (larger time gaps between tweets) but lasts longer. Second, the larger the cultural memory (Term #2) of a company, the more likely a bankruptcy event will gain High memory persistence. Bankruptcies of well-known companies, expressed by higher cultural memory, often receive widespread media coverage and publicity, and associated attention persists longer in collective memory than bankruptcies of less prominent companies. Third, we found that the likelihood of Low-persistence memory increases with the frequency of a company’s mentions pre-bankruptcy (Term #6). This suggests that increased attention pre-bankruptcy may contribute to a shorter duration of memory of a bankruptcy event. Counterintuitively, when people are highly engaged in sharing their thoughts and ideas about an event on social media, the memory of the event fades quickly^44,45^. Thus, the event will less likely be encoded into longer-term memory^44^.

## Conclusion

In this study, we pioneered the exploration of collective memory on social media in the context of corporate bankruptcy, revealing the potential to predict which companies might retain prolonged public attention after the declaration of corporate bankruptcy. Moreover, the societal relevance of our findings is significant. Understanding the dynamics of digital collective memory in corporate communication and crisis management has practical applications in corporate governance, public policy, and financial regulation.

However, caution is warranted due to the potential for social media data to reflect artifacts like bot activities and ideological ‘echo chambers’, rather than genuine public attention. This highlights the importance of ethical and social considerations in interpreting data from digital platforms. Despite these limitations, our findings offer valuable insights for developing strategies to better manage corporate crises and improve outcomes for companies facing financial distress.

Future studies could expand the scope of data collection to include X posts (tweets) that mention a company without using the ‘@' handle, comparing these results with our current findings to provide a more comprehensive view of public discourse surrounding corporate bankruptcy. Additionally, carefully assessing the relationship of consumers to corporate products (e.g., luxury goods vs. utilities) in the context of bankruptcy-related attention will further elucidate the factors influencing how the public assesses and remembers corporate bankruptcy events.

### Supplementary Information

Supplementary Information 1.

## Data Availability

All attention frequency data and supplementary data are publicly available on GitHub: https://github.com/bartala/Bankruptcy.

## Code availability

All analysis code is publicly available on GitHub: https://github.com/bartala/Bankruptcy.

## References

[1] Halbwachs, M. *On Collective Memory*, trans. FJ and VY Ditter 1950 (1992).

[2] Assmann J, Czaplicka J (1995). Collective memory and cultural identity. New German Critique.

[3] Candia C, Jara-Figueroa C, Rodriguez-Sickert C, Barabasi A-L, Hidalgo CA (2019). The universal decay of collective memory and attention. Nat. Hum. Behav..

[4] Rubin DC (1995). Memory in Oral Traditions: The Cognitive Psychology of Epic, Ballads, and Counting-out Rhymes.

[5] Buskell A (2017). What are cultural attractors?. Biol. Philos..

[6] Roediger HL, DeSoto KA (2016). Recognizing the presidents: Was alexander hamilton president?. Psychol. Sci..

[7] Hirst W, Yamashiro JK, Coman A (2018). Collective memory from a psychological perspective. Trends Cogn. Sci..

[8] Coman A, Hirst W (2015). Social identity and socially shared retrieval-induced forgetting: The effects of group membership. J. Exp. Psychol. Gener..

[9] Yu AZ, Ronen S, Hu K, Lu T, Hidalgo CA (2016). Pantheon 1.0, a manually verified dataset of globally famous biographies. Sci. Data.

[10] West R, Leskovec J, Potts C (2021). Postmortem memory of public figures in news and social media. Proc. Natl. Acad. Sci..

[11] Jara-Figueroa C, Yu AZ, Hidalgo CA (2019). How the medium shapes the message: Printing and the rise of the arts and sciences. PloS ONE.

[12] Higham KW, Governale M, Jaffe AB, Zulicke U (2017). Fame and obsolescence: Disentangling growth and aging dynamics of patent citations. Phys. Rev. E.

[13] Assmann J (2011). Communicative and cultural memory. Cultural Memories: The Geographical Point of View.

[14] Mena S, Rintamaki J, Fleming P, Spicer A (2016). On the forgetting of corporate irresponsibility. Acad. Manag. Rev..

[15] Crane A (2013). Modern slavery as a management practice: Exploring the conditions and capabilities for human exploitation. Acad. Manag. Rev..

[16] Fig D (2005). Manufacturing amnesia: Corporate social responsibility in south africa. Int. Affairs.

[17] Fine G (2012). Sticky Reputations: The Politics of Collective Memory in Midcentury America.

[18] Brockmeier J (2002). Remembering and forgetting: Narrative as cultural memory. Cult. Psychol..

[19] Zadek S (2004). On civil governance. Development.

[20] McDonnell M-H, King B (2013). Keeping up appearances: Reputational threat and impression management after social movement boycotts. Admin. Sci. Quart..

[21] Storm BC, Bjork EL, Bjork RA (2012). On the durability of retrieval-induced forgetting. J. Cogn. Psychol..

[22] Desai VM (2014). The impact of media information on issue salience following other organizations’ failures. J. Manag..

[23] Garcıa-Gavilanes R, Mollgaard A, Tsvetkova M, Yasseri T (2017). The memory remains: Understanding collective memory in the digital age. Sci. Adv..

[24] Mestyan M, Yasseri T, Kertesz J (2013). Early prediction of movie box office success based on wikipedia activity big data. PloS ONE.

[25] Yasseri T, Bright J (2016). Wikipedia traffic data and electoral prediction: Towards theoretically informed models. EPJ Data Sci..

[26] Garcıa-Gavilanes R, Tsvetkova M, Yasseri T (2016). Dynamics and biases of online attention: the case of aircraft crashes. R. Soc. Open Sci..

[27] Kim Y, Weon BM (2021). Stretched exponential dynamics in online article views. Front. Phys..

[28] Setiowati, D. A., Zainal, A. G., Kartika, T. & Aryanti, N. Y. Public relation strategy in handling Bank Lampung “bankrupt” reporting issues. *Int. J. Multidis. Res. Publ. (IJMRAP)* **4**(8) (2022).

[29] Hong H, Lim T, Stein JC (2000). Bad news travels slowly: Size, analyst coverage, and the profitability of momentum strategies. J. Finance.

[30] Doukas JA, McKnight PJ (2005). European momentum strategies, information diffusion, and investor conservatism. Eur. Financ. Manag..

[31] Igarashi N, Okada Y, Sayama H, Sano Y (2022). A two-phase model of collective memory decay with a dynamical switching point. Sci. Rep..

[32] Dawkins MC, Bhattacharya N, Bamber LS (2007). Systematic share price fluctuations after bankruptcy filings and the investors who drive them. J. Financ. Quant. Anal..

[33] Morse D, Shaw W (1988). Investing in bankrupt firms. J. Finance.

[34] Satopaa, V., Albrecht, J., Irwin, D. & Raghavan, B. Finding a “kneedle” in a haystack: Detecting knee points in system behavior. In: *2011 31st Int. Conf. Distrib. Comput. Syst. Worksh.* 166–171 (IEEE, 2011).

[35] Feller W (1948). On the kolmogorov-smirnov limit theorems for empirical distributions. Ann. Math. Stat..

[36] Hutto, C. & Gilbert, E. VADER: A parsimonious rule-based model for sentiment analysis of social media text. In: *Proc. Int. AAAI Conf. Web Social Media*. **8,** 216–225 (2014).

[37] Bartal A, Pliskin N, Tsur O (2020). Local/global contagion of viral/non-viral information: Analysis of contagion spread in online social networks. Plos ONE.

[38] Wang D, Song C, Barabasi A-L (2013). Quantifying long-term scientific impact. Science.

[39] Rubin DC, Wenzel AE (1996). One hundred years of forgetting: A quantitative description of retention. Psychol. Rev..

[40] Massey FJ (1951). The kolmogorov-smirnov test for goodness of fit. J. Am. Stat. Assoc..

[41] Coelho LMS (2015). Bad news does not always travel fast: Evidence from chapter 11 bankruptcy filings. Account. Finance.

[42] Xing FZ, Cambria E, Welsch RE (2018). Intelligent asset allocation via market sentiment views. IEEE Comput. Intell. Mag..

[43] Khan, W. *et al.* Stock market prediction using machine learning classifiers and social media, news. *J. Amb. Intell. Human. Comput.* 1–24 (2020)

[44] Tamir DI, Templeton EM, Ward AF, Zaki J (2018). Media usage diminishes memory for experiences. J. Exp. Soc. Psychol..

[45] Schacter DL (2022). Memory sins in applied settings: What kind of progress?. J. Appl. Res. Memory Cogn..

