# Supplementary material for: Persistence of collective memory of corporate bankruptcy events discussed on X (Twitter) is influenced by pre-bankruptcy public attention
Source: Sci Rep. 2024 Mar 19;14:6552. doi: 10.1038/s41598-024-53758-x (PMC10951345; doi:10.1038/s41598-024-53758-x)
Supplement: Supplementary file 1 — Supplementary Information 1. [file 41598_2024_53758_MOESM1_ESM.pdf]

Persistence of Collective Memory of Corporate Bankruptcy Events Discussed on X (Twitter) Is Influenced by Pre-Bankruptcy Public Attention

Kathleen M. Jagodnik[1,2,3], Sharon Dekel[2,3], Alon Bartal[1*]

[1*]The School of Business Administration, Bar-Ilan University, Ramat Gan, 5290002, Israel.
[2]Department of Psychiatry, Harvard Medical School, Boston, 02129-4522, Massachusetts, USA.
[3]Department of Psychiatry, Massachusetts General Hospital, Boston, 02129-4522, Massachusetts, USA.

*Corresponding author(s). E-mail(s): alon.bartal@biu.ac.il;
Contributing authors: kjagodnik@mgh.harvard.edu;
sdekel@mgh.harvard.edu;

Supplementary information. Supplementary Methods A, Supplementary Figures B1, B2, B3, Supplementary Table B1

Appendix A Supplementary Methods

A.1 Identify and remove outliers

In R, the `boxplot` function uses the "1.5 x IQR" rule to identify and remove outliers. This rule defines an outlier as any observation that falls more than 1.5 times the interquartile range (IQR) below the first quartile (Q1) or above the third quartile (Q3) of the data. In more detail, the function first calculates the median of the data (the horizontal line inside the box). Then, the function calculates the first and third quartiles of the data (the lower and upper edges of the box, respectively). The function calculates the interquartile range (IQR), which is the difference between the third and first quartiles. The function extends "whiskers" from the box to the highest and lowest data points that are within 1.5 times the IQR of the box. These whiskers are represented by the vertical lines outside the box. Any data points that fall outside the

whiskers are considered outliers. To remove outliers from the data, we use the boxplot 'out' output (outliers) to filter the data.

A.2 Long-term Boost Histogram

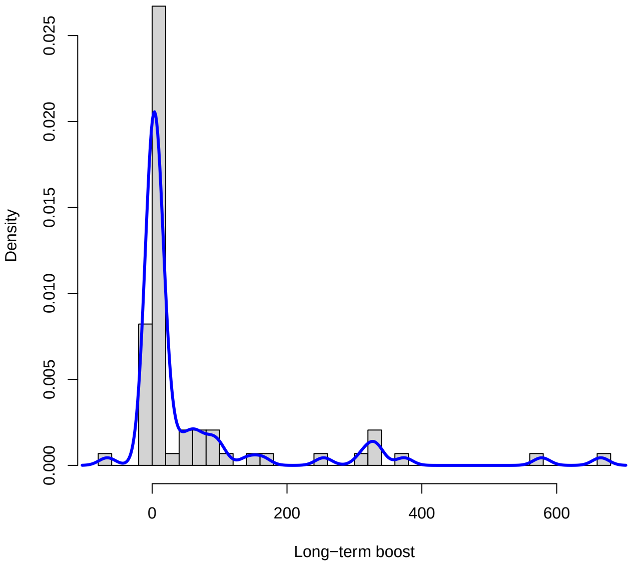

Fig. A1: Distribution of Long-Term Boost.

A.3 Companies with Low or High Level of Persistent Bankruptcy Memory on Twitter

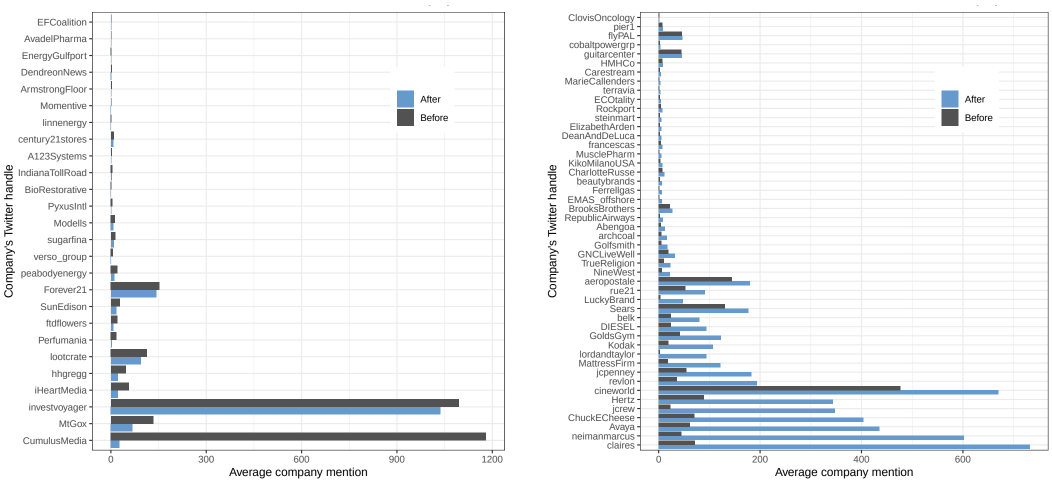

(a) Companies with Low level of persistent bankruptcy memory on Twitter.

(b) Companies with High level of persistent bankruptcy memory on Twitter.

Fig. A2: Companies with (a) Low or (b) High persistence level of memory ordered by the difference between the average number of mentions before and after bankruptcy. For the 'After' period, we calculated the average number of mentions in the Long-Term period. For the 'Before' period, we calculated the average daily mentions before bankruptcy announcement (i.e., the Pre-Announcement Mean).

A.4 Memory Model Fitting

We fit five models (Biexponential, Exponential, Hyperbolic, Logarithmic, and Power) for companies in each memory persistence level (Low and High); in total, we fit 10 models.

A.4.1 R Code for Model Fitting for Low Level of Persistence Memory about Bankruptcy Events

```
Call:
lm(formula = y ~ exp(x), data = forgotten)

Residuals:
    Min      1Q  Median      3Q     Max
-43.27  -34.16  -17.62   10.16  186.60

Coefficients:
              Estimate Std. Error t value Pr(>|t|)
(Intercept)  7.300e+01  9.920e+00   7.359 4.16e-08 ***
exp(x)      -7.285e-12  4.806e-12  -1.516     0.14
---
Signif. codes:  0 '***' 0.001 '**' 0.01 '*' 0.05 '.' 0.1 ' ' 1

Residual standard error: 53.27 on 29 degrees of freedom
Multiple R-squared:  0.07341,   Adjusted R-squared:  0.04146
F-statistic: 2.298 on 1 and 29 DF,  p-value: 0.1404


Call:
lm(formula = y ~ log(x + 0.01), data = forgotten)

Residuals:
     Min       1Q   Median       3Q      Max
-37.061  -12.339   -4.702    3.738   68.238

Coefficients:
              Estimate Std. Error t value Pr(>|t|)
(Intercept)    140.893      8.212   17.16  < 2e-16 ***
log(x + 0.01)  -31.783      3.028  -10.50 2.17e-11 ***
---
Signif. codes:  0 '***' 0.001 '**' 0.01 '*' 0.05 '.' 0.1 ' ' 1

Residual standard error: 25.26 on 29 degrees of freedom
Multiple R-squared:  0.7917,   Adjusted R-squared:  0.7845
F-statistic: 110.2 on 1 and 29 DF,  p-value: 2.168e-11
```

```
Formula: y ~ a/(1 + b * x)

Parameters:
    Estimate Std. Error t value Pr(>|t|)
a  2.596e+02  7.637e+01   3.399  0.00198 **
b -4.290e+07  4.263e+14   0.000  1.00000
---
Signif. codes:  0 '***' 0.001 '**' 0.01 '*' 0.05 '.' 0.1 ' ' 1

Residual standard error: 76.37 on 29 degrees of freedom

Number of iterations to convergence: 36
Achieved convergence tolerance: 1.49e-08


Formula: y ~ a1 * exp(-b1 * x) + a2 * exp(-b2 * x)

Parameters:
    Estimate Std. Error t value Pr(>|t|)
a1 158.98842   29.16246   5.452 9.07e-06 ***
b1   0.76830    0.30806   2.494  0.01905 *
a2 109.12311   20.87660   5.227 1.66e-05 ***
b2   0.04597    0.01303   3.527  0.00153 **
---
Signif. codes:  0 '***' 0.001 '**' 0.01 '*' 0.05 '.' 0.1 ' ' 1

Residual standard error: 23.03 on 27 degrees of freedom

Number of iterations to convergence: 28
Achieved convergence tolerance: 1.49e-08


Formula: y ~ a * (x + 1e-04)^(-b)

Parameters:
  Estimate Std. Error t value Pr(>|t|)
a 86.35726    7.51680  11.489 2.59e-12 ***
b  0.12592    0.01446   8.707 1.38e-09 ***
---
Signif. codes:  0 '***' 0.001 '**' 0.01 '*' 0.05 '.' 0.1 ' ' 1

Residual standard error: 36.3 on 29 degrees of freedom

Number of iterations to convergence: 10
```

```
Achieved convergence tolerance: 6.745e-06
```

A.4.2 R Code for Model Fitting for High Level of Persistence Memory about Bankruptcy Events

```
Call:
lm(formula = y ~ exp(x), data = Unforgotten)

Residuals:
     Min       1Q   Median       3Q      Max
 -28.342  -16.444   -9.740    0.626  153.412

Coefficients:
              Estimate Std. Error t value Pr(>|t|)
(Intercept)  5.161e+01  7.107e+00   7.262 5.37e-08 ***
exp(x)      -1.223e-12  3.443e-12  -0.355    0.725
---
Signif. codes:  0 '***' 0.001 '**' 0.01 '*' 0.05 '.' 0.1 ' ' 1

Residual standard error: 38.16 on 29 degrees of freedom
Multiple R-squared:  0.004334,  Adjusted R-squared:  -0.03
F-statistic: 0.1262 on 1 and 29 DF,  p-value: 0.7249


Call:
lm(formula = y ~ log(x + 0.01), data = Unforgotten)

Residuals:
     Min       1Q   Median       3Q      Max
 -24.058  -13.031   -1.139    9.007   65.988

Coefficients:
              Estimate Std. Error t value Pr(>|t|)
(Intercept)     98.519      6.501   15.15 2.58e-15 ***
log(x + 0.01)  -21.043      2.397   -8.78 1.16e-09 ***
---
Signif. codes:  0 '***' 0.001 '**' 0.01 '*' 0.05 '.' 0.1 ' ' 1

Residual standard error: 20 on 29 degrees of freedom
Multiple R-squared:  0.7267,    Adjusted R-squared:  0.7172
F-statistic: 77.09 on 1 and 29 DF,  p-value: 1.157e-09


Formula: y ~ a/(1 + b * x)

Parameters:
```

```
    Estimate Std. Error t value Pr(>|t|)
a  2.050e+02  5.279e+01   3.883 0.000548 ***
b -6.766e+07  9.283e+14   0.000 1.000000
---
Signif. codes:  0 '***' 0.001 '**' 0.01 '*' 0.05 '.' 0.1 ' ' 1

Residual standard error: 52.79 on 29 degrees of freedom

Number of iterations to convergence: 37
Achieved convergence tolerance: 1.49e-08


Formula: y ~ a1 * exp(-b1 * x) + a2 * exp(-b2 * x)

Parameters:
     Estimate Std. Error t value Pr(>|t|)
a1 183.475012  11.856218  15.475 6.04e-15 ***
b1   0.548725   0.076340   7.188 9.91e-08 ***
a2  28.612564   5.602989   5.107 2.29e-05 ***
b2  -0.016299   0.009192  -1.773   0.0875 .
---
Signif. codes:  0 '***' 0.001 '**' 0.01 '*' 0.05 '.' 0.1 ' ' 1

Residual standard error: 11.76 on 27 degrees of freedom

Number of iterations to convergence: 14
Achieved convergence tolerance: 1.49e-08


Formula: y ~ a * (x + 1e-04)^(-b)

Parameters:
  Estimate Std. Error t value Pr(>|t|)
a 63.39833    4.51890   14.03 1.86e-14 ***
b  0.13069    0.01152   11.34 3.51e-12 ***
---
Signif. codes:  0 '***' 0.001 '**' 0.01 '*' 0.05 '.' 0.1 ' ' 1

Residual standard error: 21.69 on 29 degrees of freedom

Number of iterations to convergence: 8
Achieved convergence tolerance: 2.716e-06
```

A.5 Sentiment Analysis

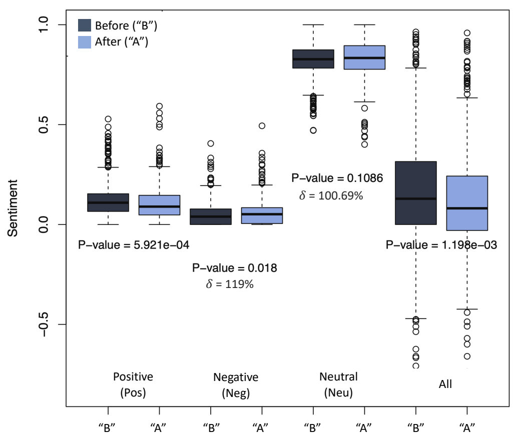

(a) Sentiment of Low persistent memory of bankruptcy event.

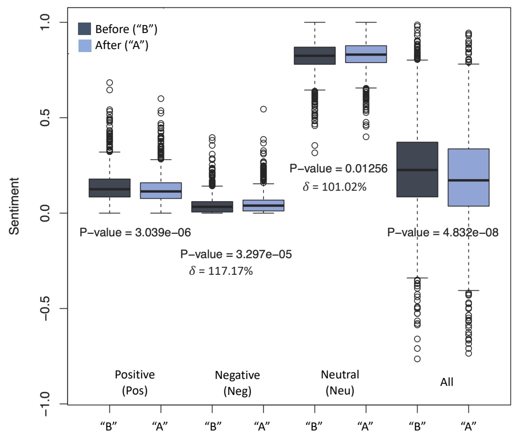

(b) Sentiment of High persistent memory of bankruptcy event.

Fig. A3: Boxplots of sentiment before and after bankruptcy announcement in (a) Low and (b) High memory persistence level. For Low and High persistence, we present the positive (Pos), negative (Neg), neutral (Neu), and the overall (All) compound score metric that calculates the sum of all the lexicon ratings (Pos, Neg, and Neu) that have been normalized between -1 (most extreme negative) and +1 (most extreme positive). For events with Low persistence (Fig. A3a), we find significant differences in sentiments before vs. after bankruptcy for Pos, Neg, and All (pairwise Wilcoxon tests, P-values $= 5.921e^{-4}, 0.018$, and 0.001, respectively). Neutral sentiment before vs. after bankruptcy was found insignificant for Low-persistence events. For events with High persistence (Fig. A3b) we find significant differences in sentiments before vs. after bankruptcy for Pos, Neg, Neu, and All (P-value $= 3.039e^{-6}, 3.297e^{-5}, 0.012$, and $4.832e^{-8}$), respectively. Additionally, we computed the percentile change (denoted δ) of Neg sentiment before vs. after bankruptcy for both Low and High persistence levels. For Neg sentiment, the δ of High-persistence memory is significantly lower (p-value $= 8.038e^{-9}$) than the δ of Low-persistence memory. For Neu sentiment, the δ of High-persistence memory is significantly higher (p-value $= 1.768e^{-6}$) than the δ of Low-persistence memory. Hence, the percentile change for High-persistence memory is higher for Neu and lower for Neg sentiment compared with Low-persistence memory. Boxplot features: Center line, median; box limits, upper and lower quartiles; whiskers, 1.5x interquartile range; points, outliers.

A.6 Persistence Prediction

Logistic regression summary

```
Call:
NULL

Coefficients:
                          Estimate  Std. Error  z value  Pr(>|z|)
(Intercept)               2.349134   0.907483    2.589
0.00964 **
avg_time_from_first      -0.231819   0.070656   -3.281
0.00103 **
Pre_announcement_mean    -0.136948   0.065033   -2.106
0.03522 *
number_of_tweets_before   0.003665   0.001691    2.167
0.03025 *
---
Signif. codes:  0 '***' 0.001 '**' 0.01 '*' 0.05 '.' 0.1 ' ' 1

(Dispersion parameter for binomial family taken to be 1)

    Null deviance: 84.473  on 64  degrees of freedom
Residual deviance: 56.598  on 61  degrees of freedom
AIC: 64.598
```

Model performance

```
# ------------ 10-fold Cross Validation ----------------
Confusion Matrix and Statistics

          Reference
Prediction  0  1
         0 39  8
         1  3 15

               Accuracy : 0.8308
                 95% CI : (0.7173, 0.9124)
    No Information Rate : 0.6462
    P-Value [Acc > NIR] : 0.0008541

                  Kappa : 0.6108

 Mcnemar's Test P-Value : 0.2278000

            Sensitivity : 0.9286
            Specificity : 0.6522
         Pos Pred Value : 0.8298
```

```
         Neg Pred Value : 0.8333
              Precision : 0.8298
                 Recall : 0.9286
                     F1 : 0.8764
             Prevalence : 0.6462
         Detection Rate : 0.6000
   Detection Prevalence : 0.7231
      Balanced Accuracy : 0.7904

       'Positive' Class : 0


# ---------- Evaluation over the Test set ---------------


Confusion Matrix and Statistics

          Reference
Prediction  0  1
         0 13  4
         1  2  4

               Accuracy : 0.7391
                 95% CI : (0.5159, 0.8977)
    No Information Rate : 0.6522
    P-Value [Acc > NIR] : 0.2604

                  Kappa : 0.3894

 Mcnemar's Test P-Value : 0.6831

            Sensitivity : 0.8667
            Specificity : 0.5000
         Pos Pred Value : 0.7647
         Neg Pred Value : 0.6667
              Precision : 0.7647
                 Recall : 0.8667
                     F1 : 0.8125
             Prevalence : 0.6522
         Detection Rate : 0.5652
   Detection Prevalence : 0.7391
      Balanced Accuracy : 0.6833
```

```
'Positive' Class : 0
```

Appendix B Supplementary Table B1

Table B1: Companies analyzed in this study.

ID	Company	Twitter Username	Public/ Private	Followers	Following	Bankruptcy Date
1	A123 Systems	A123Systems	Public	1844	185	Oct. 16, 2012
2	Abengoa	Abengoa	Public	19813	357	Feb. 24, 2016
3	Aeropostale	aeropostale	Public	515531	588	May 4, 2016
4	Arch Coal	archcoal	Public	3664	346	Jan. 11, 2016
5	Armstrong Flooring	ArmstrongFloor	Public	10561	1089	May 8, 2022
6	Avadel Pharmaceuticals	AvadelPharma	Public	1179	251	Feb. 6, 2019
7	Avaya Holdings Corporation	Avaya	Private	38925	4690	Jan. 19, 2017
8	Beauty Brands	beautybrands	Private	8403	707	Jan. 6, 2019
9	Belk	belk	Private	96045	218	Feb. 23, 2021
10	BioRestorative Therapies	BioRestorative	Public	1422	389	Mar. 20, 2020
11	Brooks Brothers	BrooksBrothers	Private	65444	197	July 8, 2020
12	Carestream Health	Carestream	Private	11323	1864	Aug. 23, 2022
13	Century 21 Stores	century21stores	Private	14128	7947	Sep. 10, 2020
14	Charlotte Russe	CharlotteRusse	Private	72100	9438	Feb. 4, 2019
15	Chuck E. Cheese	ChuckECheese	Private	46105	415	Jun. 25, 2020
16	Cineworld Group	cineworld	Public	303261	16975	Sep. 7, 2022
17	Claire's Holdings LLC	claires	Private	65400	225	Mar. 19, 2018
18	Clovis Oncology, Inc.	ClovisOncology	Public	1121	4	Dec. 11, 2022
19	Cobalt International Energy	cobaltpowergrp	Public	223	498	Dec. 14, 2017
20	Cumulus Media	CumulusMedia	Public	5472	1146	Nov. 29, 2017
21	Dean & DeLuca	DeanAndDeLuca	Private	20044	1990	Mar. 31, 2020
22	Dendreon	DendreonNews	Public	2539	1006	Nov. 10, 2014
23	Diesel S.p.A.	DIESEL	Private	179000	737	Mar. 5, 2019
24	ECOtality	ECOtality	Public	2088	828	Sep. 16, 2013
25	Elizabeth Arden, Inc.	ElizabethArden	Public	84859	2295	Jun. 15, 2022
26	Energy Future Holdings	EFCoalition	Private	737	925	Apr.29, 2014

27	Ezra Holdings Ltd.	EMAS_offshore	Public	1071	470	Mar. 18, 2017
28	Ferrellgas Partners LP	Ferrellgas	Private	1913	163	Jan. 11, 2021
29	Forever 21	Forever21	Private	1900000	351	Sep. 29, 2019
30	Francesca's Holdings Corporation	francescas	Public	14802	1145	Dec. 3, 2020
31	FTD / FTD Flowers	ftdflowers	Private	12800	21	Jun. 3, 2019
32	GNC Holdings, Inc.	GNCLiveWell	Public	187956	3972	Jun. 23, 2020
33	Gold's Gym	GoldsGym	Private	108910	498	May 4, 2020
34	Golfsmith	Golfsmith	Private	16779	556	Sep. 14, 2016
35	Guitar Center	guitarcenter	Private	226845	2135	Nov. 21, 2020
36	Gulfport Energy Corporation	EnergyGulfport	Public	146	20	Nov. 13, 2020
37	Hertz Global Holdings, Inc.	Hertz	Public	75694	32589	May 22, 2020
38	HHGregg	hhgregg	Public	31800	900	Mar. 6, 2017
39	Houghton Mifflin Harcourt	HMHCo	Private	18203	2928	May 21, 2012
40	IHeartMedia, Inc.	iHeartMedia	Public	23588	820	Mar. 14, 2018
41	Indiana Toll Road Concession Company	IndianaTollRoad	Private	5100	131	Sep. 21, 2014
42	J. Crew	jcrew	Private	326482	987	May 4, 2020
43	J.C. Penney	jcpenney	Public	421867	63	May 15, 2020
44	Kiko Milano USA	KikoMilanoUSA	Private	4270	47	Jan. 11, 2018
45	Kodak	Kodak	Public	72785	1	Jan. 19, 2012
46	Linn Energy	linnenergy	Public	1914	89	May 11, 2016
47	Loot Crate	lootcrate	Private	455892	4809	Aug. 11, 2019
48	Lord & Taylor	lordandtaylor	Private	60844	897	Aug. 2, 2020
49	Lucky Brand	LuckyBrand	Private	22882	1527	July 3, 2020
50	Marie Callender's	MarieCallenders	Private	4397	1543	Jun. 13, 2011
51	Mattress Firm Holding Corp.	MattressFirm	Private	47200	554	Oct. 5, 2018
52	Modell's Sporting Goods	Modells	Private	21717	5225	Mar. 11, 2020

53	Momentive Performance Materials Inc.	Momentive	Private	1300	35	Apr.13, 2014
54	Mt. Gox	MtGox	Private	22200	1	Feb. 28, 2014
55	MusclePharm	MusclePharm	Public	309707	29	Dec. 15, 2022
56	Neiman Marcus	neimanmarcus	Private	355716	1601	May 7, 2020
57	Nine West Holdings Inc.	NineWest	Private	45400	387	Apr.6, 2018
58	Peabody Energy	peabodyenergy	Public	9184	709	Apr.13, 2016
59	Perfumania Holdings, Inc.	Perfumania	Public	19000	159	Aug. 26, 2017
60	Philippine Airlines	flyPAL	Public	2985854	1	Sep. 4, 2021
61	Pier 1 Imports	pier1	Public	105976	1253	Feb. 17, 2020
62	Pyxus International, Inc.	PyxusIntl	Public	643	29	Jun. 15, 2020
63	Republic Airways	RepublicAirways	Private	4597	234	Feb. 25, 2016
64	Revlon, Inc.	revlon	Public	454428	512	Jun. 15, 2022
65	Rockport	Rockport	Private	6294	940	May 14, 2018
66	Rue21, Inc.	rue21	Private	32400	1091	May 16, 2017
67	Sears Holdings Corp.	Sears	Public	198100	9293	Oct. 15, 2018
68	Stein Mart	steinmart	Public	5882	1188	Aug. 12, 2020
69	Sugarfina	sugarfina	Private	22300	172	Sep. 6, 2019
70	SunEdison	SunEdison	Public	13246	5195	Apr.21, 2016
71	TerraVia Holdings, Inc.	terravia	Public	466	101	Aug. 2, 2017
72	True Religion	TrueReligion	Private	43803	189	July 5, 2017
73	Verso Corporation	verso_group	Public	142	183	Jan. 26, 2016
74	Voyager Digital Ltd.	investvoyager	Public	163996	1151	July 6, 2022
